# Parenting involvement of Thai expectant fathers during the last trimester pregnancy of their spouses exhibiting depressive symptoms: a qualitative study

**DOI:** 10.1186/s12884-025-07288-7

**Published:** 2025-02-17

**Authors:** Pranee C. Lundberg, Nitikorn Phoosuwan

**Affiliations:** 1 Department of Public Health and Caring Sciences, Uppsala University, Box 564, Uppsala, SE-751 22 Sweden; 2 Department of Community Health, Kasetsart University Chalermphrakiat Sakon Nakhon Province Campus, Sakon Nakhon, Thailand

**Keywords:** Expectant fathers, Late pregnancy, Parenting involvement, Qualitative method, Thailand

## Abstract

**Background:**

Becoming a father is a challenging process that starts during pregnancy. During the late pregnancy period, depressive symptoms are the most common mental disorders among women. Engaged fatherhood is a strategy for gender equality that is good for the health outcomes of men, their spouses and their children. This study aims to explore the involvement of Thai expectant fathers during the last trimester of their partners’ pregnancy when the mothers exhibit depressive symptoms.

**Methods:**

A qualitative study with semi-structured interviews was conducted by use of an interview guide. All data were transcribed and subjected to content analysis.

**Results:**

Twenty-seven expectant fathers at seven antenatal care clinics of public hospitals in a province of north-eastern Thailand participated. Six categories emerged: Having mixed emotional feelings, Adapting to spouse’s emotional mood swings, Getting into challenges of fatherhood, Taking increased responsibility in the role of father, Receiving social support, and Wishing improvement of maternity care service. The expectant fathers experienced different psychological and physical situations and received support from family members and social networks, but they had not been encouraged to engage in parental education. Some were satisfied with healthcare service and some would like to be more involved.

**Conclusions:**

On the basis of these findings, it is suggested that an intervention program should be developed that aims to engage expectant fathers in maternity health services. This could be done most effectively by starting with men who wish themselves to be involved in the healthcare of their spouses and continuing with men who do not have such wish. Professional support for nurses/midwives should be not only informative but also emotional and practical when needed.

## Background

Becoming a father is a challenging process that starts during pregnancy. Transition into fatherhood is a significant experience of expectant fathers (EFs) that comes with major biological, psychological and sociocultural changes for many of them [1, 2]. Expectant fathers may have to take on several roles during the transition [3, 4] such as those of fathers and providers. Experience of a gap between their expectations and their realities may give rise to combined feelings, such as being both happy and worried about their transition [4, 5]. During pregnancy, fathers express uncertainty and exclusion [6]. Previous experiences from childbirth may facilitate preparation and strengthen a sense of security [2, 7]. Social support from their social network can give EFs a feeling of calmness and security for their offspring and parenthood [8]. Guidance with useful and accurate information may help to create their fatherhood identity and strengthen their fatherhood role [9].

Parental involvement [10] comprises three parts: engagement, accessibility, and responsibility. Engagement concerns the father’s direct contact with his child through activities together. Accessibility refers to the father’s availability for interaction with his child and his presence for the child. Responsibility refers to the role of the father in ensuring that his child’s needs are taken care of, e.g., by providing financial and emotional support for the child and participating in decisions concerning the child [10]. A father’s involvement during pregnancy influences positively the health outcomes for himself, his spouse and his child [3, 11], and plays a vital role in the safety of his spouse’s pregnancy and childbirth by ensuring access to care and provision of emotional and financial support [12]. Fathers want to take part in preparations for parenthood and be able to support their spouses [13, 14]. However, fathers often feel ignored by healthcare professionals (HCPs) even though they want to be involved from the beginning of pregnancy [14].

In Asia, the role of breadwinner is important for men’s identities, while their involvement in caregiving activities is low compared with that of men in Western societies [15]. Fathers have been expected to be leaders, protectors and providers of their families, whereas mothers have been expected to be housewives and to take care of their children according to Thai tradition [16].

In Thailand, antenatal care (ANC) in a normal pregnancy is provided free of charge by nurses/midwives in public-health services. A pregnant woman may be seen by an obstetrician once during antenatal care. Obstetricians, gynecologists, and nurses/midwives are responsible for women with high-risk pregnancies. All pregnant women should have at least four antenatal care visits. However, the number of antenatal visits varies depending on the woman and her healthcare providers [17]. According to the standard guideline of the World Health Organization [18], the woman attends antenatal check-ups every month until 28 weeks of gestation, then every fortnight during weeks 28 to 32, and finally every week after week 32.

During the antenatal period, depressive symptoms are the most common mental disorders among pregnant women and their partners [19]. Thai adolescent fathers try to manage the father’s role and to avoid unplanned parenthood [16]. If they receive more support from their social networks, they will have increased ability to manage their transition into fatherhood [20]. In Thailand, governmental campaigns have introduced parenting classes to prepare parents for their new role and to encourage men to be involved in child rearing [21]. Fathers’ experiences and needs during pregnancy and childbirth have been studied mainly in Western countries [22, 23].

Pregnancy and parenthood are challenging with many emotional, physical and social changes to the mother, her partner and their surroundings [24]. Perinatal resilience is multifaceted and includes individual, social and environmental systems helping to cope with challenges, stressors, and adversities in life through personal growth, family balance, adaptation, or acceptance [25]. Social support, sense of mastery, self-efficacy, and self-esteem enhance the capacity to be resilient and probably prevents mental health problems [24, 25]. Depressive symptoms among expectant mothers may cause depression in spouses, developmental problems for infants, and maternal suicide [19]. The prevalence of depressive symptoms among pregnant women during their second and third trimesters in northwestern rural China has been found to be 16.14% which is higher than findings in Chinese urban areas [26]. In a rural area of north-eastern Thailand, the prevalence of antenatal depressive symptoms among pregnant women in their late pregnancy was found to be 46.8% in 2017 [27], while in an urban area the prevalence of such symptoms of women in the similar stage of pregnancy was found to be 18.9% in 2019 [28]. Associated factors were low income, limited social support, young women, low psychological well-being, extended family and marital conflicts [27, 28]. Some women have reported the importance of their partners’ support for their well-being and sense of self-esteem [29]. More paternal emotional support and involvement during pregnancy is associated with less maternal anxiety, stress, and depression [30]. Social and cultural norms determine the roles of fathers [31]. The men had no desire to be more actively involved in antenatal care and delivery beyond their roles as providers [32]. There is difference between health system’s policy for male involvement and societal expectations [33].

Previous studies have focused on experiences of first-time fathers during their spouses’ pregnancies [4, 7, 8, 9, 14, 16, 20, 22]. Expectant fathers can experience stress due to negative feelings about pregnancy, role restrictions related to parenthood, fear of childbirth, social isolation and self-efficacy about infant care [24]. As far as we know, only few studies have focused on how EFs (both first-time fathers and experienced fathers) support their spouses exhibiting antenatal depressive symptoms. If EFs have more engagement and equity in childcare, they may help to compensate for the mental health problems of their spouses. Nurses/midwives can increase their understanding and knowledge of the transition into fatherhood of EFs in order to better prepare and involve future fathers. Therefore, the aim of this study was to explore the involvement of Thai expectant fathers during the last trimester of their partners’ pregnancy when the mothers exhibit depressive symptoms.

## Methods

### Study design

A descriptive qualitative design was used to explore and describe the direct feelings and experiences of parenting involvement during the last trimester pregnancy among EFs having spouses exhibiting depressive symptoms [34]. The focus of this research is on answering “what” and “how” questions that enable deeper understanding of the real-life of EFs [35]. This design made it possible to summarize experiences in everyday terms [36].

### Study setting

This study was carried out in Sakon Nakhon, one of the ten provinces with the lowest incomes in the northeastern Thailand during 2015–2022 and with a large number of childbirths per year. The religions (Buddhist, Christian and Islam) and residential areas (rural and urban) are mixed [37], and low income may be the reason for a high prevalence of depressive symptoms during the perinatal period [26]. Data were collected at seven hospitals with a large number of childbirths per year, one provincial hospital (2,343), one general hospital (1,178) and five hospitals (123–600) [37].

### Participants

The participating EFs were selected by using purposive sampling. The inclusion criteria were: (1) Being an expectant father at the time of recruitment and data collection, (2) having a spouse in the last trimester of pregnancy (28–37 weeks gestational age) exhibiting depressive symptoms (i.e. a score of seven or more on the Thai version of the Edinburgh Postnatal Depression Scale (EPDS) validated for Thai pregnant women [26], (3) receiving ANC service in the selected hospitals, (4) being a Thai native, and (5) being willing to participate in the study. On the basis of previous use of the Thai version of the EPDS, a cut-off score of seven was considered suitable for detection of major and/or minor depression among Thai women [38]. The exclusion criteria were (1) not living with his spouse at the time of data collection, (2) having migrated from other countries, and (3) reporting a history of mental disease. The characteristics of the participants were considered to represent current expectant fathers. In the Thai northeastern culture, men were breadwinners, while married women used to stay home and take care of children. However, some women migrated, leaving their children under the care of their relatives or parents. Men felt that it was their main duty to earn money and take care of their family. The couples returned to their hometown during pregnancy and childbirth [39].

### Data collection

A semi-structured interview guide with six open-ended questions concerning feelings and experiences of parenting involvement among EFs during the late pregnancy of their spouses was developed from previous studies [23, 26]. These questions were as follows: (a) How have you been feeling about parenting involvement during your wife’s/partner’s pregnancy? (b) What are your experiences of the new life situation you will soon get with a newborn baby? (c) What do you think would help (have helped) you cope with your situation? (d) What roles do you think expectant parents should have during the pregnancy period? (e) What kinds of support and services do you need during your wife’s/partner’s pregnancy? (f) Is there something else that you would like to tell me or that I should know? Background information was requested at the end of the interview guide. The guide was pilot-tested on two EFs in a nearby province and was slightly adjusted before use.

Nurses/midwives at each hospital provided a list of EFs having their spouses in the last trimester of pregnancy for individual interview. Expectant fathers with a wide range of ages, religions and residential areas were asked if they were willing to participate in the in-depth interviews. They chose the place, e.g. a room in a hospital or at home, and the time for the interviews. Both authors were Thai natives with experience of qualitative methods. The first author (nursing, midwifery, PhD-degree) took notes about facial expressions and gestures during the interviews. The second author (public health, PhD-degree), having the same gender as the EFs, carried out individual interviews in Thai to make the participants feel comfortable when describing their experiences. Each interview lasted about 60 min and was recorded digitally. After the interview, each participant received a summary of his answers and had the opportunity to clarify any ambiguities. All participants confirmed the summary. Thirty-two EFs from the hospitals were contacted and invited to participate. Recruitment was finished when nothing significantly new had emerged in the last few interviews. All interviews were transcribed verbatim in Thai by the second author. Then the transcripts were rechecked by the first author.

### Data analysis

Data from the interviews and notes were subjected to inductive qualitative content analysis [34]. The aim was to attain a condensed and broad description of phenomena, and the outcome of the analysis was concepts or categories describing these phenomena [40]. Therefore, this method was expected to be well suited for analyzing the multifaceted parenting involvement of EFs during the last trimester pregnancy of their spouses exhibiting depressive symptoms. The authors analyzed the data separately, and then checked and discussed the findings together. The transcripts were read repeatedly to obtain overall sense and make reflective notes. The analysis began by highlighting sentences of importance and dividing them into meaning units. The meaning units were condensed and labelled with 59 short codes. Then, the codes were compared to identify similarities and differences. Categories were developed on the basis of codes that included manifest content (visible and obvious components). Emerging categories and sub-categories were tested and revised through analyses of the interviews, and then the results were discussed and modified to ensure reliability. See Table 1. Two Thai external peer experts of qualitative research and the subject checked and validated emerging codes, sub-categories and categories. Disagreements were discussed until consensus was reached [40]. The final results were fed back to the nurses/midwives at the district and provincial levels in order to provide information to frontline workers and policy makers.

**Table 1:** Examples of the analytical process

Meaning units	Condensed meaning unit	Codes	Sub-categories	Categories
- I felt very glad and excited when my girlfriend told me about her pregnancy even though we were not ready to have a child.… I wish to see my baby’s face soon.	Feel glad when knowing about girlfriend’s pregnancy	Positive feelings	Being glad, excited and happy	Having mixed emotional feelings
- When my partner told me about her pregnancy…. I had to work extra to earn more money to prepare for childbirth, travel to hospital and medicine.	Have to work extra to earn more money to prepare for childbirth	Economics	Responsibility as a breadwinner	Taking increased responsibility in the role of father
- I touch to feel the movement of my fetus before going to bed every day. I and my wife turn on Thai songs from the mobile phone for our fetus to listen	Touch to feel the movement of fetus every day and turn on music for the fetus to listen	Touching and turning on music	Bonding with the fetus	Getting into challenges of fatherhood

Trustworthiness was addressed in several steps: credibility, confirmability, dependability, and transferability [41]. Credibility was achieved by interviewing participants with varied characteristics in terms of age, civil status, education, occupation, and types of EFs. It was strengthened by letting the participants check their answers at the end of the interviews and have the opportunity to clarify any ambiguities. The interview guide was tested before use to make it clear. This led to replacement of a few words. The results of the study were discussed in seminars with participation of other researchers. In addition, two Thai external peer experts in qualitative methods checked and validated the results. Confirmability was strengthened through transcriptions by the second author and rechecking by the first author. Also, the data were analyzed separately by the authors, and reliability was ensured by discussing and modifying the findings. Dependability was increased by use of tested interview guides, field notes and separate analyses. Transferability was strengthened by providing thorough descriptions of the contents and methods.

### Ethical approval

Approval was given by Ethics Committees in Sweden (Dnr 2016/544) in December 2016 and in Thailand (SWDCPH2017-003) in January 2017. Data were collected from the beginning of July to the end of August, 2017. The participants were fully informed about the purpose of the study, assured of anonymity and confidentiality, and told that anyone could drop out at any time. They had given informed consent verbally and in writing. Alphabetical codes were used to identify the transcribed data. The study was conducted in accordance with the Declaration of Helsinki [42].

## Results

Twenty-seven first-time and experienced EFs (consent rate 84.4%) accepted to participate in the study. After the twenty-seventh interview, the researchers found that nothing new emerged from the interviews. Their age range was 17 to 50 years, and they were agriculturists or daily workers. Their demographic characteristics are presented in Table 2. Five EFs did not participate because they did not have time. One experienced EF lived with the expectant mother during the perinatal period and experienced the birth of his child. The number of previous children of the experienced fathers was from 1 to 5 (average number = 2.46), and the ages of their children varied from four to 15 years (average ages = 8.77 years).

**Table 2:** Demographic characteristics of expectant fathers during the last trimester pregnancy of their spouses with depressive symptoms (*N* = 27)

Characteristics	Number (%)
Age (Median = 29; range from 17 to 50 years old)	
- < 20 years	2 (7.4)
- 20 years and more	25 (92.6)
Religion	
- Buddhist	25 (92.6)
- Christian	2 (7.4)
Marital status	
- Married	20 (74.0)
- Live together	7 (26.0)
Education	
- Secondary school or lower	18 (66.7)
- High school or more	9 (33.3)
Occupation	
- Daily work	13 (48.2)
- Agriculture	7 (25.9)
- Self-employment	7 (25.9)
Type of family	
- Extended family	19 (70.4)
- Nuclear family	8 (29.6)
Years of living together (Median = 3; range from 1–20	
years)	
- Less than 5 years	16 (59.2)
- 5 years or more	11 (40.7)
Residential area	
- Rural - Urban	15 (55.6) 12 (44.4)
Type of expectant fathers	
- First time	14 (51.8)
- Experienced	13 (48.2)

Six categories of parenting involvement among the EFs during the last trimester pregnancy of their spouses exhibiting depressive symptoms were identified: Having mixed emotional feelings, Adapting to spouse’s emotional mood swings, Taking increased responsibility in the role of father, Getting into challenges of fatherhood, Receiving social support, and Wishing improvement of maternity care service. See Table 3.

**Table 3:** Categories and subcategories of parenting involvement of expectant fathers during the last trimester pregnancy of their spouses with depressive symptoms

Categories	Subcategories
• Having mixed emotional feelings	- Being glad, excited and happy - Anxious, worried and fearful
• Adapting situations towards spouses’ emotional mood swings	
• Getting into challenges of fatherhood	- Bonding with the fetus - Expectation of baby’s gender
• Taking increased responsibility in the role of father	- Responsibility as a breadwinner - Helping with daily routines and household
• Receiving social support	- Support from parents and parent-in-laws - Support from healthcare professionals - Support from other social networks
• Wishing improvement of maternity care service	

### Having mixed emotional feelings

Mixed emotional feelings were both positive and negative feelings. Most of the EFs had positive feelings such as gladness and happiness when they knew about their spouses’ pregnancy and prepared for transition to fatherhood. Some EFs felt excited to get a new member in their family because they had waited for a long time to have a baby or believed that they could not have a child. The expectant first-time adolescent fathers did not feel ready for fatherhood. In accord with Buddhists’ belief, however, they described that they and their spouses were against abortion. Experienced fathers felt happy to have a sibling for their child/children, while first-time fathers experienced mixed feelings of happiness, excitement and worry having a baby.

I felt very glad and excited when my girlfriend told me about her pregnancy even though we were not ready to have a child. But we don’t want to have an abortion as we are Buddhists. I wish to see my baby’s face soon.… (EF3, 18 years old).

Negative feelings were also described by EFs. Some EFs were anxious about the unknown future and about the health conditions of their spouses. They were afraid of their spouses’ complications during pregnancy such as gestational diabetes, low platelets, Thalassemia, and vaginal bleeding. Some EFs mentioned that they had been told that their spouses had a risk of gestational diabetes in the antenatal period because of high level of blood sugar and had to follow up regularly when visiting maternal health service at ANC. They felt worried that their spouses would have to deliver the baby by caesarean along with fear about the health of their fetuses.

I felt anxious and worried about her health. When my wife had been pregnant around four months, the doctor told her about high level of blood sugar that led to a risk of gestation diabetes. She was recommended to follow up regularly at ANC and not to eat sweet things.… However, the fetus had good movement. (EF13, 35 years old)

### Adapting to spouse’s emotional mood swings

The EF’s meeting with friends, their drinking of alcohol after work, and their habit of coming home late led to emotional swings of their spouses who also sometimes believed that the EFs had extra-marital sex. All EFs were faced with their spouses’ up-and down moods. Therefore, some EFs tried to keep good relationships. To avoid quarrel, they tried to change their behavior, e.g. by drinking less alcohol with their friends, staying more at home, and reducing the number of cigarettes they smoked per day. Another way to avoid quarrel was walking away and coming back when their spouses felt better.

My partner has often a bad mood, and her mood swings have increased from early pregnancy until now. She believed I had another woman and started to quarrel with me. I walked away and came back when her mood was better. (EF5, 34 years old)

### Getting into challenges of fatherhood

Most of the EFs faced the challenge of fatherhood by bonding with the fetus during their spouses’ pregnancy to give them a sense of being a father. They did this by touching their spouse so that they could feel how the fetus moved and by talking with the fetus every evening. Some EFs also turned on music from their mobile phones for the fetus, e.g. Thai songs for babies according to advice from nurses/midwives at ANC clinics.

I touch to feel the movement of my fetus before going to bed every day. I and my wife turn on Thai songs from the mobile phone for our fetus to listen. (EF6, 25 years old)

There were different expectations about the baby’s gender among the EFs, their spouses and their parents or parents-in-law. The first-time EFs wished to have a son as their first child, while some EFs described that the gender of their baby was not important to them. Some of them could get information about their baby’s gender after ultrasound examination carried out by nurses/midwives. However, some could not because of the position of the fetus, and therefore their spouses could feel depressed about the baby’s gender.

When I knew that my wife had got pregnant I wished to have a son as the first child like my parents but my wife told me that the baby’s gender did not matter. I thought my wife felt depressed when talking about our baby’s gender. (EF14, 27 years old)

### Taking increased responsibility in the role of father

Economy was an important problem that reflects all EFs’ main responsibility as breadwinners who provide financial support of their families. However, the experienced EFs mentioned that they had to work hard to get extra income for the new baby and the other children, while the adolescent first-time EFs mentioned that they received economical support from their parents and parents-in-law. Most of them had non-permanent work. They received pay per day, and their spouses did not work after getting pregnant. For these reasons, there was often not enough money for the needs of their families when moving back to their hometown. Some EFs worked extra to earn more money in order to save for delivery even though childbirth in public hospitals was free according to the Thai universal health coverage.

When my partner told me about her pregnancy…. I had to work extra to earn more money to prepare for childbirth, travel to hospital and medicine. (EF23, 41 years old)

Most of the EFs reduced the stress and depression of their spouses by in advance together buying necessary things such as clothes, diapers, and baby bed for their babies. However, some EFs did not because in accord with Thai traditional culture they believed that it would not be good to prepare things for their baby before childbirth.

I and my wife go to the department store to buy clothes, diapers, etc. for our baby. I think I have already prepared so that my wife does not feel stress. (EF10, 38 years old)

Helping EFs’ spouses with daily life and household activities was mentioned by the EFs. They helped with housework such as washing clothes, cooking, and washing dishes. They also bought food or fruits when their spouses wished to eat.

I think she finds it difficult to move her body because she is near delivery of our baby, so I help her by washing clothes and dishes when I am back home… (EF4, 22 years old).

All EFs brought their spouses to the hospital when they had an appointment for examination of gestation. Some EFs were allowed by bosses at their workplaces to accompany their spouses to ANC clinics. However, they waited outside while their spouses met with nurses/midwives for examination and participation in antenatal sessions. For their spouses’ safety, they also changed to public transport instead of using motorcycles for visits at ANC clinics. They were afraid of accidents.

I accompany my wife to the hospital every time when she has an appointment… I wait outside and bring her back home after finishing. (EF7, 36 years old)

### Receiving social support

Support from parents and parents-in-law to the EFs and their spouses during pregnancy and childbirth consisted of money, a place to stay until delivery after moving back to their hometowns, things for the newborns and help with their child/children. Some EFs mentioned that their parents-in-law would help them take care of the newborns so that they could go back to their work in industrial provinces such as Bangkok, Samutprakarn, and Chonburi. However, some EFs who were adolescents had come into conflicts with their parents-in-law when their spouses got pregnant.

My parents-in-law have told us that they will help us take care of our baby after delivery, and my partner will stay with them. I could travel back to Bangkok and work at the same place as I did before. (EF6, 25 years old)

Nurses/midwives gave information and advice to the EFs’ spouses that concerned, e.g., books, blood tests, medicine, touching and talking with the fetus, taking care of the baby, nutrition (meat, egg, fruits, and milk), iodine to prevent iodine deficiency, and exercise. Some EFs described that they got information about pregnancy from their spouses who had got it when visiting ANC clinics. Their spouses were satisfied with the antenatal care information and the maternal care service. Doctors had also given advice about sterilization to spouses with 3–4 children.

My wife has told me that she is satisfied with information and maternal care service at the ANC clinic. She has also got free milk and iodine to take at home.… The doctor recommends us sterilization after childbirth because we have already three children. (EF23, 41 years old)

Social support was also provided from social networks through which the EFs got some assistance from the local government or community. They were eligible to receive financial support for hospital care in connection with childbirth, monthly support after childbirth, and loans if needed. Expectant Fathers who did not work at government offices could get monthly support for one year for their child if they registered with the Local Administrative Office (Or Por Tor) after delivery.

I and my wife do not need to pay for the cost of childbirth because of the universal health insurance. After delivery we will get 600 Baht per month financial support from Or Por Tor (Local Administrative Office) after submitting the registration form for a newborn child. (EF6, 25 years old)

Some EFs and their spouses used the Internet to search for information (e.g. about pregnancy and related risks and complications). They also used Facebook groups for mothers. Some EFs mentioned that their neighbors had suggested what to do during pregnancy and after childbirth.

My wife usually contacted other expectant mothers via Facebook. Sometimes I help her use Google to find out what we want to know about pregnancy, e.g. taking care of herself, eating food and doing physical exercise. (EF15, 30 years old)

### Wishing improvement of maternity care service

Insufficient information about pregnancy and childbirth was expressed by some EFs. They felt excluded from first-hand information related to maternal and child health and dependent on second-hand information from their spouses who had been informed at ANC clinics. The first-time EFs believed that antenatal care attendance was for the pregnant women only. They wished to be involved in the antenatal care service and be included in parenting classes together with their spouses.

I think I am excluded… I want to be involved in information about parenthood and what I should do in my role of father. I also want to participate in the antenatal class. (EF11, 24 years old)

Some EFs described that nurses/midwives need to focus more on their work at ANC clinics instead of talking together for a long time while pregnant women had to wait for maternal care service. In addition, some EFs were dissatisfied with the regular ANC services because of long waiting times at ANC clinics, e.g. arriving at the hospital at 6 am and waiting until 11 am for examination of gestation.

I was not satisfied with the health service at the hospital… I saw nurses/midwives chatting with each other a lot while pregnant women had to wait long time for examination. (EF15, 30 years old)

## Discussion

### Emotional feelings of the EFs

The finding of both positive and negative feelings of parenting involvement among the EFs during the last trimester pregnancy of their spouses exhibiting depressive symptoms is in line with previous studies [4, 5, 6, 7]. Transition to fatherhood is an emotional roller coaster, i.e., a tension between different feelings, such as happiness and worry, about EFs’ future as fathers [6, 7]. During pregnancy, fathers express worry for their spouses and babies [4, 5] and compassion for spouses with difficulties [7]. Professional support from nurses/midwives could be not only informative but also emotional and practical when needed. Paternal involvement in parenting educational classes could also reduce maternal depression during pregnancy.

Bonding with the fetus by touching and talking is in accord with the findings by Lamb and colleagues [10] that fathers were engaged through contact, bonding and activities together with the child. Engagement by fathers can have positive impact on the psychological, behavioral, social and cognitive outcomes of the child [27]. It is important that nurses/midwives encourage EFs to participate in parenting classes that prepare for fatherhood according to a policy of the Ministry of Public Health in Thailand [21] concerning paternal involvement.

### Breadwinners and responsible for household activities

All EFs in this study took responsibility as breadwinners. In Thailand, as in many other Asian countries, the notion of a father is a leader responsible for the financial situation of his family [15]. Sakhon Nakhon is an agricultural region and a low-income province [37], and many workers get income day-by-day and need many working hours to maintain their income. The participating EFs received pay per day, which they used for the family’s costs of living even though Thailand has offered all women the opportunity to deliver in a public hospital free of charge [43]. In the Thai tradition, a man is always considered as the breadwinner who supports his family members [16]. Therefore, the first-time adolescent EFs in this study had adapted to the situation by changing their lifestyles and striving to care for their families, not only by taking on the role as breadwinner but also by taking care of the child and doing housework. Nurses/midwives need to gain a deeper understanding of EFs’ experiences to provide useful information for the transition to fatherhood. In particular, they could encourage EFs to engage in parental education classes. Policy of male involvement in pregnancy and childbirth could be introduced effectively by engaging men who are keen to be involved in the healthcare of their spouses.

The finding that the EFs helped with physical household activities and support of spouses’ wishes is in accord with findings of previous studies. Fathers have been reported to provide physical and emotional help for their spouses during childbirth and also to discuss pregnancy and childbirth with them [44]. First-time fathers have reported to modify their behavior by taking increased responsibility at home [45]. EFs have an important role in satisfying the nutritional and health needs of their spouses during pregnancy [13].

### Effects of Thai culture during pregnancy

In this study, drinking alcohol after work was a reason for quarrel between the EFs and their spouses. Pregnant women sometimes had difficult relationships with their partners because of these partners’ alcohol consumption [46]. In the Thai society, men use alcohol for recreation and as a way to facilitate interaction, and this behavior is a part of the culture also in northeastern Thailand [47]. Use of alcohol can lead to inappropriate behavior among men, such as violence in the form of verbal abuse when quarrelling, which may influence expectant mothers’ mental well-being [32]. It might be difficult for the individual man to change his drinking behavior as the consumption of alcohol is a widely accepted social activity in Thailand. Also, extramarital sex was found to be a reason for conflicts. Thai men are widely perceived as having a driving need of sex, whereas women are viewed as having control of their sexual feelings. The habit of extramarital sex seems to be common among Thai men [48]. Sexuality is linked with the social construction of gender, and the expectations among men and women in the society can lead to serious repercussions and family break ups. Therefore, nurses/midwives should inform future EFs about pregnant women’s emotional mood and about paternal education to avoid conflicts with their spouses caused by habits of alcohol consumption and extramarital sex. Talking about sexual behavior, with focus on expectant parents, might need more attention during ANC visits.

The finding that abortion was not selected when the first-time adolescent EFs and their spouses knew about pregnancy even though they were not ready for parenthood could be related to their religious belief. Buddhists believe in rebirth and teach that individual human life begins at conception. Many Buddhists feel that abortion is prohibited because it involves the deliberate ending of a life [49].

Male gender of the baby was wished by some EFs, particularly first-time fathers. This might be one of the causes for the depressive symptoms of their spouses. Thai culture is similar to Chinese culture which has long favored sons over daughters, and this cultural preference of sons still exists [50]. Therefore, sons are more valued than daughters, and only production of male progeny is viewed as a continuation of the family line.

### Support to perform paternal roles

The finding that the EFs in this study received social support that helped them perform their paternal roles is consistent with previous studies [5, 8, 44]. Family support is essential for coping with the transition to fatherhood, and Asian culture places great emphasis on extensive family support [16, 20]. It is a cultural norm that women in northeastern Thailand move to their in-law family after marriage, and after childbirth most women mainly take care of their children with support from EFs’ family [47]. Also, the finding that EFs received help from local government or community is in line with previous studies. Currently, Thailand offers two different leave benefits for new fathers: government employees have a 15-day leave from work and other employees get a monthly allowance for a child during the first six-year period [21]. The EFs felt comforted when they received help from their families and other sources [7, 43]. Professional support from nurses/midwives could aim at strengthening social support to plan for EFs’ involvement.

### Parenting involvement in the transition to fatherhood

The benefits of fatherhood involvement are not limited to children’s and mothers’ wellbeing; there is growing evidence documenting the benefits of fatherhood involvement for the men themselves [11]. The findings of lack of involvement in antenatal care, insufficiency of information, and dissatisfaction with the maternal care service are in accord with several studies. Fathers feel invisible in the pregnancy process [51], and they feel excluded from parental education classes [8, 23]. Fathers who are involved during pregnancy and childbirth are likely to develop their parental identity earlier than fathers who are not [11]. Fathers experience a lack of information for themselves as fathers [14]. Therefore, when giving pregnancy-related information, nurses/midwives need to act as advisors of EFs [51]. According to the policy of father involvement in parental education classes of the Ministry of Public Health in Thailand, nurses/midwives need to provide parental education classes that prepare EFs for their roles as fathers and involvement in child rearing. Therefore, health policy makers need to formulate and implement policies that meet the needs of EFs.

Fathers’ involvement is important during pregnancy. Therefore, nurses should increase the awareness of the importance of father involvement during pregnancy, encourage fathers to participate in prenatal visits and ask questions, and educate fathers on the pregnancy process and the procedures of prenatal care. This could be achieved by preparing an intervention for the transition to fatherhood at ANC directed to groups of future EFs. The intervention should comprise self-reflection, mindfulness practices, group discussions, and support programs.

### Strengths and limitations of this study

This study was conducted by use of qualitative method to obtain rich data for understanding of issues related to parenting involvement of Thai EFs during the last trimester pregnancy of their spouses having depressive symptoms. The in-depth interviews gave increased understanding of the EFs’ experiences through their own words. Furthermore, this study focused on both first time and experienced fathers; therefore, the results reflect parenting involvement of expectant fathers in general. The results are valuable as a basis for further investigations aimed at improving parental education and engaging EFs in parental education classes in accord with the policy of father involvement of the Thai Ministry of Public Health. However, the study was carried out in a province of north-eastern Thailand which might limit the transferability to other settings. In addition, data were collected in 2017 but the characteristics of the participants are considered to represent current expectant fathers. Another limitation is that the interview questions did not ask deeply about the EFs’ violence towards their pregnant spouses. In this study, the EDPS was not used to screen depressive symptoms among EFs. Therefore, further study may be needed.

## Conclusions and implications

The EFs emphasized their responsibilities as breadwinners of the family and as receivers of social support. They were dissatisfied with the maternal care service and wished to be involved at ANC clinics. They also felt the need to be acknowledged, seen and encouraged by nurses/midwives. On the basis of these findings, it is suggested that an intervention program should be developed that aims to engage expectant fathers in maternity health services. This could be done most effectively by starting with men who wish themselves to be involved in the healthcare of their spouses and continuing with men who do not have such wish. When providing parental education classes, male involvement could be encouraged by including and educating EFs and preparing them for their roles as fathers and their involvement in child rearing. Professional support from nurses/midwives should be not only informative but also emotional and practical when needed.

## Data Availability

The datasets used and/or analyzed during the current study are available from the corresponding author on reasonable request.

## Abbreviations

ANC: Antenatal Care
EFs: Expectant Fathers
EPDS: Edinburgh Postnatal Depression Scale
